# Linking Ecosystem Service Supply and Demand to Evaluate the Ecological Security in the Pearl River Delta Based on the Pressure-State-Response Model

**DOI:** 10.3390/ijerph20054062

**Published:** 2023-02-24

**Authors:** Wei Liu, Jinyan Zhan, Yongbo Zhai, Fen Zhao, Michael Asiedu Kumi, Chao Wang, Chunyue Bai, Huihui Wang

**Affiliations:** 1 College of Geography and Environment, Shandong Normal University, Jinan 250358, China; 2 State Key Laboratory of Water Environment Simulation, School of Environment, Beijing Normal University, Beijing 100875, China; 3 Shandong Provincial Institute of Territorial Space Planning, Jinan 250014, China; 4 School of Resources and Environmental Engineering, Ludong University, Yantai 264025, China; 5 School of Labor Economics, Capital University of Economics and Business, Beijing 100070, China

**Keywords:** ecological security, obstacle factors, ecosystem service supply and demand, pressure–state–response model, Pearl River Delta

## Abstract

The increase in population and economic development has made environmental issues more serious and threatens regional ecological security and sustainable development. Currently, most indicators in the related research field of ecological security tend to be socio-economic and neglect depicting the state of the ecosystems. This study, therefore, assessed the ecological security by constructing the evaluation index system embedded in the ecosystem service supply and demand based on the pressure–state–response model and identified the key obstacles to ecological security in the Pearl River Delta from 1990 to 2015. Our results showed that soil retention, carbon sequestration, and water yield increased with fluctuation except for grain production and habitat quality. The grain demand, carbon emission, and water demand increased sharply by 10.1%, 769.4%, and 17.5%, respectively. The ecosystem service supply areas were mainly located in the low hills, while the demand regions were mainly in the low plain areas. The ecological security index’s decline in vitality was caused by the decrease in the pressure index, indicating that the ecological security showed an inevitable deterioration and increased pressure on the ecosystem. During the study period, the source of the five key obstacle factors changed from the state layer and response layer to the pressure layer. The accumulative degree of the five top obstacle factors was above 45%. Therefore, governments should grasp the key indicators to improve ecological security as this study provides the theoretical basis and scientific information for sustainable development.

## 1. Introduction

Ecological security theories and assessment methods are being constantly improved. As problems increasingly threaten the ecosystem and environment, people have begun to pay attention to ecological security in the 1970s [1]. Ecological security was put forward in the 1980s after the Chernobyl nuclear power plant accident in the former Soviet Union [2]. In a broad sense, it refers to the condition that human life, health, well-being, fundamental rights, life security sources, necessary resources, social order, and human ability to adapt to environmental changes are not threatened [3]; in a more specific sense, it refers to the security of natural and semi-natural ecosystems, that is, the overall reflection level of ecosystem integrity [4].

Research on ecological security mainly dates back to 1990, with the theoretical basis of ecological security gradually expanding to ecological security patterns, temporal and spatial evolution, and the driving forces of ecological security and ecological security early warning [5]. Ecological security assessment methods or models include the comprehensive index method [6], ecological model, and landscape ecology [7]. Specifically, they mainly include the pressure–state–response model (PSR) and its extended model (e.g., DPSIR model) [8], fuzzy multi-attribute decision-making and analytic hierarchy process [9], ecological footprint method [10], minimum resistance model [11], landscape structure analysis [12], and the ecosystem service value [13]. Different models have varied advantages and disadvantages. In comparison, the PSR model can better represent the complex relationship between social, economic, and natural systems, and identify the main driving factors of ecological security change. The primary research paradigm of ecological security is to evaluate its state by constructing the corresponding index system according to the study area. However, most indicators tend to be socio-economic and neglect to depict the state of the ecosystems.

Ecological security is closely related to ecosystem services [14]. Ecological security is the guarantee of sustainable development, while ecosystem services are the representation of ecological security. Ecosystem service is the core of ecological security evaluation, reflecting the core position of human security [15]. Ecological security evaluation based on ecosystem services has gradually become a new hot spot and direction. Many achievements have been made from the perspective of the research progress of ecosystem services and ecological security. However, there are few studies on embedding ecosystem service indicators into the evaluation index system [16]. Most studies have only measured ecological security from the aspects of landscape ecosystem structure and functional security. Ecological security assessment considering ecosystem services is still in the exploratory stage. Huang et al. [2] evaluated the ecological security state of Xiamen based on the quality and quantity of ecosystem services. Zhang et al. [17] identified landscape ecological security patterns in the Beijing–Tianjin–Hebei region from the perspective of the supply and demand of ecosystem services. Lyu et al. [18] constructed and optimized the ecological security pattern based on the ecosystem services of the Liaohe River Basin. However, most of these studies have focused on the spatial layout of ecological security rather than the state of ecological security. Ecological security mainly refers to the security of natural ecosystems and semi-natural ecosystems. Ecosystem services, for example, food supply, water supply, and carbon sequestration, reflect the supply capacity of different ecosystems, that is, the state of the ecosystem. In contrast, the ecosystem services demand of human society (e.g., grain demand, water consumption, and CO_2_ emissions) reflects the demand level for ecosystem services, that is, human society’s pressure on the ecosystem. Embedding the supply and demand of ecosystem services into the PSR model is significant for the scientific evaluation of regional ecological security and provides more objective and accurate basic decision-making information for ecological management.

The Pearl River Delta (PRD) is one of the urban agglomerations, and is the core zone of the urbanization strategy in China. In 2015, the PRD generated 9.04% of the national economy with only 0.6% of the land area and about 4.27% of the population. During 1990 and 2015, construction land increased about 4576.87 km^2^, while the arable and forest lands decreased by about 3356.81 and 1196.92 km^2^, respectively. The expansion of construction land and the development of urbanization has caused ecological problems such as a decline in soil quality, which places great pressure on the regional ecological security. The ecological security level has declined continuously in recent years, in particular, the central areas show a poor situation [19]. The construction of the Guangdong–Hong Kong–Macao Greater Bay Area is a new development engine for the PRD, so the ecosystems in the PRD may be under greater pressure than before. Therefore, exploring the ecological security path and state from the ecosystem service supply and demand perspective is essential for high-quality development and regional sustainability.

In this study, we aimed to (1) analyze the supply and demand of key ecosystem services in the PRD from 1990 to 2015; (2) evaluate the ecological security based on the index system embedded in the ecosystem service supply and demand; and (3) identify the obstacle factors of ecological security and propose policy implications. This study provides a theoretical basis and scientific information for sustainable development.

## 2. Materials and Methods

### 2.1. Study Area

**Figure 1:**
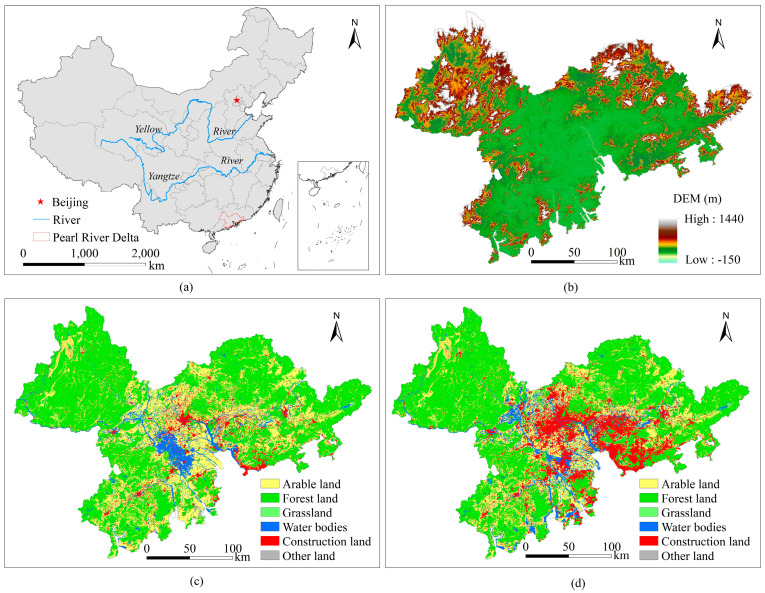
Geographic location (**a**), digital elevation model (**b**), and land use types in 1990 (**c**) and 2015 (**d**) for the Pearl River Delta.

PRD is located in the southeast of Guangdong Province in China, covering an area of 54,754 km^2^ (Figure 1a). Its annual average temperature is between 21 and 22 °C, and precipitation is between 1600 and 2000 mm. Mountains and hills are mainly distributed in the east, north, and west areas, while low plain areas are distributed in the central part (Figure 1b). Since the implementation of China’s reform and opening-up, the urbanization and economy of the PRD have developed rapidly. The GDP increased from RMB 100.7 billion in 1990 (as the base value) to RMB 2839.3 billion in 2015, with an annual growth rate of RMB 109.54 billion. The PRD had a permanent population of 58.7 billion in 2015, nearly 2.48 times as many in 1990. Alongside the increase in economy and population, the PRD is faced with problems such as tight resource and energy constraints and increasing ecological and environmental pressure.

From 1990 to 2015, the land use in the PRD underwent significant changes, which mainly manifested as a reduction in arable land and forest land, the expansion of construction land, and the basic stability of other land use types (Figure 1c,d). During the study period, the arable land decreased from 15,775.79 km^2^ to 12,420.10 km^2^ with fluctuations. The area covered by forests shrunk from 29,985.38 km^2^ to 28,788.97 km^2^. The area under construction expanded from 2908.48 km^2^ to 7482.80 km^2^ (Table 1). Little changed in the areas of grassland, water bodies, and other land. The grassland and other land decreased by 24.6 km^2^ and 66.85 km^2^, respectively, and water bodies increased by 76.13 km^2^.

### 2.2. Data Sources and Processing

The data used in this study mainly included land use data, night light data, meteorological data, socio-economic data, NDVI data, soil property data, and topography data (DEM). The land use and DEM data with 1 km × 1 km resolution were sourced from the Data Center for Resources and Environment Sciences. The socio-economic data including grain production, GDP, and proportion of coal consumption originated from local Statistical Yearbooks of the prefecture-level cities. Water consumption was derived from the Guangdong Water Resource Bulletin. Soil property data were derived from the Harmonized World Soil Database provided by the National Cryosphere Desert Data Center. The annual NDVI in the PRD at 250 m resolution was obtained from Advanced Very High-Resolution Radiometer (AVHRR) datasets of the National Oceanic and Atmospheric Administration. Night light data were sourced from the Defense Meteorological Satellite Program’s Operational Line scan System (DMSP-OLS) stable nighttime light (SNL) data and the Suomi National Polar-orbiting Partnership (NPP) Visible Infrared Imaging Radiometer Suite (VIIRS) composite data. The National Meteorological Science Data Center of China provided the meteorological data, which included daily rainfall, daily sunshine hours, and daily minimum/maximum temperatures for 25 surface meteorological observation stations in the PRD. These data were then transformed into 1 km × 1 km gridded meteorological data using conventional Kriging. The bilinear interpolation approach was used in this study to converge the data to a resolution of 1 km due to the disparate spatial resolution of the various datasets.

### 2.3. Methods

#### 2.3.1. Ecosystem Service Supply Evaluation

According to the previous related studies, we selected the key ecosystem services grain production, water yield, carbon sequestration, soil conservation, and habitat quality. We used GIS technology, the Revised Universal Soil Loss Equation (RUSLE), the Carnegie–Ames–Stanford Approach (CASA), a water balance model, and integrated valuation of ecosystem services and trade-offs (InVEST) to quantify these ESs. The methods are introduced in Table 2 in detail.

#### 2.3.2. Ecosystem Service Demand Evaluation

Grain demand. Based on high-resolution remote sensing images and night light data, we extracted the boundary line between urban and rural areas in the PRD and calculated the grain demand according to the per capita grain consumption of the urban and rural residents in the Guangdong Statistical Yearbook over the years.

(1) $$D_{grain}=\sum_{i=1}^{2}Dpg_{i}\times \rho_{i}$$

where $D_{grain}$ is the total demand of grain (kg); $Dpg_{i}$ denotes the grain demand per capita of rural (i=1) or urban residents (kg/person); $\rho_{i}$ is the urban or rural population.

Water demand. The water demand types mainly included agricultural water demand, industrial water demand, domestic water demand, and ecological environment water demand [25].

(2) $$D_{water}=D_{agr}+D_{ind}+D_{pub}+D_{res}+D_{eco}$$

where $D_{water}$ is the total water demand (m^3^); $D_{agr}$, $D_{ind}$, $D_{pub}$, $D_{res}$, and $D_{eco}$ denote agriculture, industry, urban public, residents’ life, and eco-environment water demand, respectively (m^3^).

Carbon emission. We used the carbon emission factor methods [26] to calculate the CO_2_ emissions caused by fossil energy consumption at prefectural level cities.

(3) $$E_{co2}=\sum E_{i}\times EF_{i}$$

where $E_{co2}$ is the total CO_2_ emissions; $E_{i}$ denotes the consumption of i fossil fuel; $EF_{i}$ is the emission factors of different types of fossil fuels sourced from Zhan et al. [27].

#### 2.3.3. Ecological Security Evaluation

**Figure 2:**
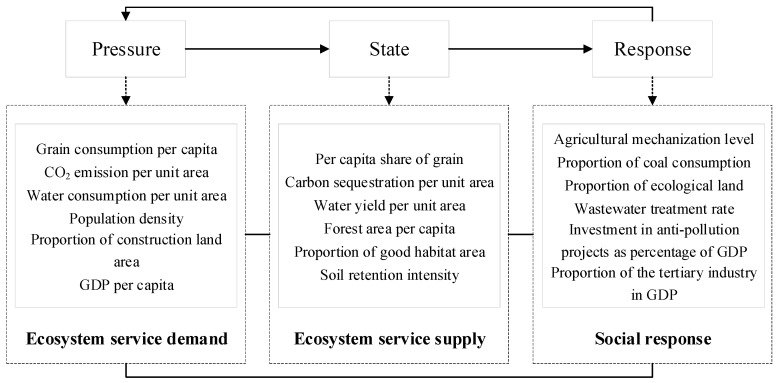
The evaluation framework of the ecological security embedded in the supply and demand of ecosystem services.

The PSR was jointly proposed by the Organization of Economic Cooperation and Development (OECD) and the United Nations Environment Program (UNEP) in the 1980s [28]. Its guiding principle is the mutual interaction between the socio-economic system and the natural ecosystem. Human beings acquire natural resources from ecosystems for survival and development, and discharge pollution into the ecosystems during production and consumption, affecting the quantity and quality of the ecosystem services. To improve the ecosystem state, governments and stakeholders can take some measures to deal with the related environmental and ecological problems. In the PSR model, the term “pressure” refers to the demands of human for ecological resources or the direct loads imposed by natural disasters on ecosystems; the term “state” denotes the capacity or current condition of a natural ecosystem to supply resources and ecosystem services; and “response” refers to the actions taken by people to lessen the effects of human activities on the ecosystems. Therefore, the supply and demand of ecosystem services were embedded into the PSR model (Figure 2).

The data were standardized by the z-score standardized methodology according to the effect of the different indices on ecological security. The entropy method was used to determine the weight of different indicators [29]. The indicator attributes and weights are shown in Table 3.

#### 2.3.4. Obstacle Factor Identification

Identifying the key obstacles to ecological security can provide scientific information to policymakers to improve ecological security [30]. Therefore, we used the obstacle degree model to investigate the obstacle factors in the PRD over time.

(4) $$P_{i,j}=1-Y_{ij}$$

(5) $$O_{ij}=\frac{P_{ij}\times W_{i}}{\sum_{i=1}^{m}P_{ij}\times W_{i}}\times 100\%$$

where $O_{ij}$ denotes the obstacle degree on the *i*th indicator in year *j*; $P_{ij}$ represents the deviation degree of indicator *i* in year *j*; $W_{i}$ is the weight of indicator *i*; m is the number of indicators; $Y_{ij}$ denotes the normalized value of indicator *i* in year *j*.

## 3. Results

### 3.1. Ecosystem Service Supply from 1990 to 2015 in the Pearl River Delta

**Figure A1:**
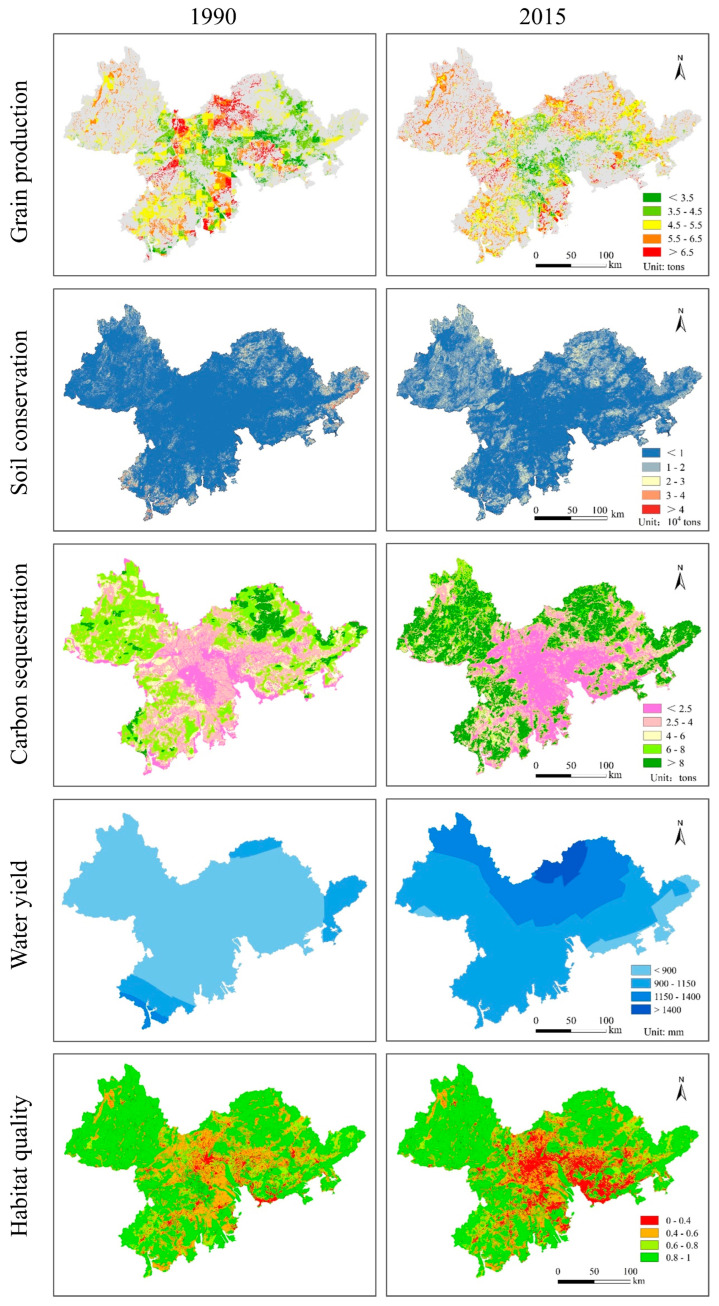
Spatial distribution of the ecosystem services supply in 1990 and 2015 in the Pearl River Delta, (Source: Liu et al., 2022 [24]).

Grain production decreased sharply from 6.98 million tons in 1990 to 3.47 million tons in 2015, with an annual reduction of 0.14 million tons per year (Table 4). This was mainly due to the expansion of construction land, which occupied a lot of arable land. During the study period, the arable land area decreased by about 3355.7 km^2^, while the construction land increased by 4574.3 km^2^. The soil retention increased from 24.30 billion tons in 1990 to 42.13 billion tons in 2015 with fluctuation (Table 4). The change trends were closely related to rainfall erosivity and vegetation cover factors. The carbon sequestration fluctuated slightly, rising from 91.45 million tons in 1990 to 111.43 million tons in 2000, then decreasing to 101.59 million tons in 2015 (Table 4). Climate factors aside from forest and grassland areas mainly affected the change in carbon sequestration. The water yield showed an increasing trend from 41.8 billion m^3^ in 1990 to 63.6 billion m^3^ in 2015 (Table 4). The habitat quality decreased continuously; the habitat quality index fell from 0.7738 to 0.7234 (Table 4). Due to the spatial distribution of precipitation varying significantly between years, the water yield did not show apparent characteristics in the spatial distribution. In addition to the water yield, the other four ecosystem services showed a similar feature in the spatial distribution: lower in the central plain areas and higher in the mountain areas (Figure A1).

### 3.2. Ecosystem Service Demand from 1990 to 2015 in the Pearl River Delta

**Figure A2:**
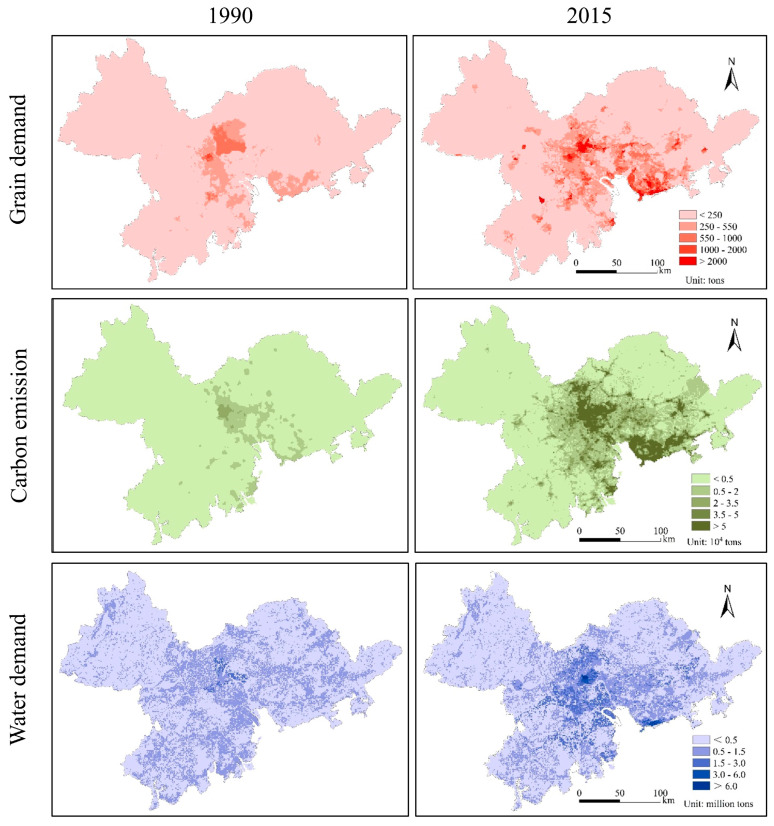
Spatial distribution of the ecosystem services demand in 1990 and 2015 in the Pearl River Delta.

The ecosystem service demand increased with fluctuation in the PRD from 1990 to 2015 (Table 5). Although grain consumption per capita decreased, the grain demand increased from 5.75 million tons to 6.33 million tons, fluctuating from 1990 to 2015 due to the rapid population increase. The water demand fluctuated from 19.42 billion tons to 22.82 billion tons. Due to economic development, CO_2_ emissions rose sharply from 69.90 million tons to 607.72 million tons. The central plain areas for the demand for ecosystem services was higher, while the low mountains and hills were lower (Figure A2). During the study period, the density and scope of the ecosystem service demand increased significantly in the central plain areas, which put tremendous pressure on the ecosystems.

### 3.3. Change Trends of the Ecological Security in the Pearl River Delta from 1990 to 2015

We used the entropy method to determine the weight of different indices (Table 3). The results show that the weights of the pressure, state, and response layers were 0.321, 0.347 and 0.332, respectively, indicating that the state layers had more impact on the ecological security than the other two layers. The percentage of coal consumption (R2) from the response layers, followed by soil retention intensity (S6), water yield per unit area (S3), GDP per capita (P6), and carbon sequestration per unit area (S2) had the greatest impact on ecological security; in contrast, the percentage of ecological land (R3) had the least significant weight.

#### 3.3.1. Pressure Index

**Figure 3:**
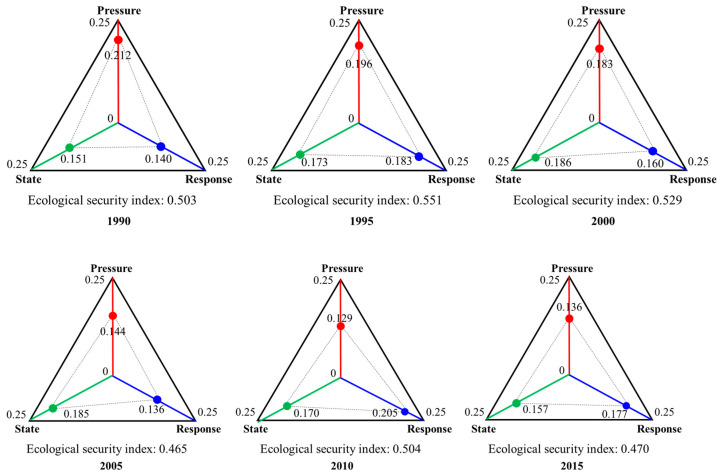
Change trends of ecological security, pressure, state, and response index in the Pearl River Delta from 1990 to 2015.

From 1990 to 2015, the ecological security pressure index showed a downward trend as a whole, decreasing from 0.212 in 1990 to 0.136 in 2015 (Figure 3), indicating that the ecological pressure of the PRD urban agglomeration kept increasing (negative indicator, the smaller the value, the greater the ecological pressure). The intensity of human disturbance to the ecosystem has increased. The changes in the pressure index can be divided into two stages. The first stage was from 1990 to 2010, where the pressure index showed a stable downward trend with an average annual decline rate of 1.56%. The main reason for the change was the rapid increase in GDP per capita, the CO_2_ emissions per unit area, and population density. In the second stage, the stress index showed an upward trend from 2010 to 2015, with an average annual growth rate of 0.942%, mainly caused by decreased food consumption per capita and water consumption per unit area.

#### 3.3.2. State Index

From 1990 to 2015, the ecological security state index showed an overall increasing trend, increasing from 0.151 in 1990 to 0.157 in 2015 (Figure 3), indicating that the ecological state of the PRD kept getting better (positive indicator, the larger the value, the better the ecological state), which was caused by the increasing supply capacity of ecosystem services. From 1990 to 2015, the capacity of ecosystem services such as water production per unit area, carbon sequestration per unit area, and soil conservation intensity gradually increased. The changes in the status index could also be divided into two stages. The first stage was from 1990 to 2000, where the status index showed a trend of rapid increase, and the average annual increase rate was 2.34%; the change was caused by the enhancement in the ecosystem service capacity such as carbon sequestration per unit area. In the second stage, the status index showed a downward trend from 2005 to 2015, with an average annual decline rate of 1.54%, caused by the decline in per capita food availability, water yield per unit area, and the proportion of high-quality habitat area.

#### 3.3.3. Response Index

From 1990 to 2015, the ecological security response index showed fluctuation characteristics. It showed an overall trend of increase, increasing from 0.140 in 1990 to 0.177 in 2015 (Figure 3), indicating that the government and society are gradually increasing their awareness of ecosystem change and environmental quality. This is reflected in the proportion of coal, the rate of wastewater treatment, the ratio of environmental pollution control in GDP, and the proportion of tertiary industry in GDP. The response index was the highest in 2010 (0.205), followed by 1995 and 2015.

#### 3.3.4. Ecological Security Index

Although the reduced scope was not very large, it also indicates that ecological security in the PRD is in a slow downtrend. From 1990 to 2015, the ecological security index showed a downward trend with fluctuation, decreasing from 0.503 in 1990 to 0.470 in 2015 (Figure 3), indicating that the ecological security of the PRD showed an inevitable deterioration as population growth and socio-economic development placed too much pressure on natural ecosystems. From the perspective of the contribution degree of each subsystem to ecological security, the difference in the contribution degree of each subsystem was slight, about 0.3. From the contribution degree of various indicators, the proportion of coal, soil and water conservation intensity, water production per unit area, per capita GDP, carbon sequestration per unit area, and other indicators had a more significant contribution to ecological security.

### 3.4. Identification of Key Obstacle Factors

**Figure 4:**
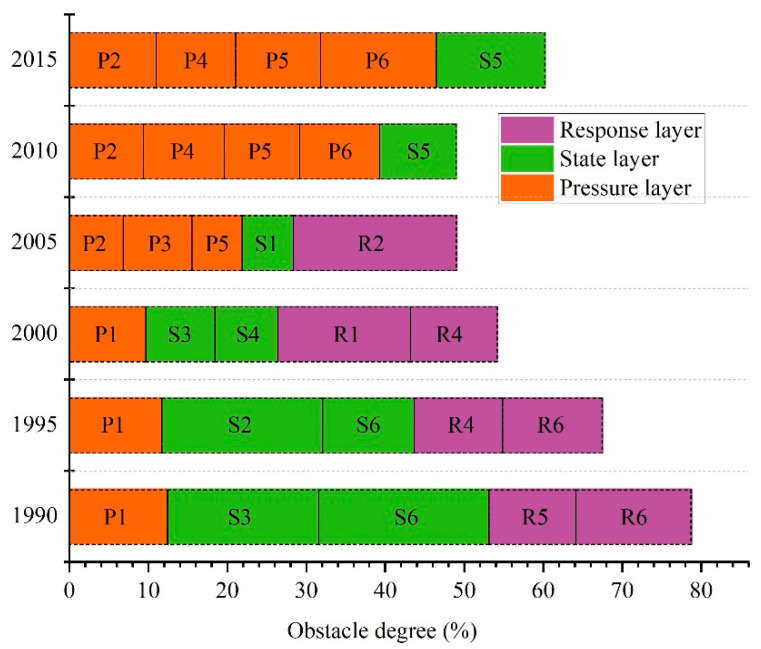
The obstacle degree of the top five key barriers to ecological security in the Pearl River Delta from 1990 to 2015.

To improve the ecological security of urban agglomeration, we identified the key obstacles in different years. The key obstacle factors of ecological security varied in various stages (Figure 4). In 1990 and 1995, most key obstacle factors were from the state and response layers. The first and most important obstacle factors mainly came from the state layer. The soil retention intensity per unit area (S6) and water yield per unit area (S3) were the barriers in 1990, and their total obstacle degree achieved 40.73%. In 1995, the key barriers in the state layer were carbon sequestration per unit area (S2) and soil retention intensity (S6), and the degree reached 31.96%. The second most important obstacle factor originated from the response layer. The proportion of the tertiary industry in GDP (R6) and investment in anti-pollution projects as a percentage of GDP (R5) had a total degree of 25.62% in 1990, while the obstacle degree of GDP per capita (R6) and population density (R4) in 1995 reached 23.82%. Grain consumption per capita (P1) was identified as the key barrier in the pressure layer in 1990 and 1995, and the obstacle degree was 12.45% and 11.75%, respectively. In 2000, the key obstacle factors were the agricultural mechanization level (R1), wastewater treatment rate (R4), grain consumption per capita (P1), water yield per unit area (S3), and forest area per capita (S4). In addition to the carbon sequestration per unit area (S2), the key barriers in 2005 had not been identified before 2000 including the proportion of coal consumption (R2), water consumption per unit area (P3), CO_2_ emissions per unit area (P2), and the per capita share of grain (S1). In 2010 and 2015, the main obstacle factors affecting ecological security were from the pressure layer including the density (P4), GDP per capita (P6), the proportion of construction land area (P5), and CO_2_ emissions per unit area (P2). The total obstacle degree of the above four factors was as high as 39.35% and 46.53% in 2010 and 2015, respectively. The proportion of good habitat area (S5) in the state layer also significantly impacted ecological security in 2010 and 2015, where the degree reached 9.69% and 13.72%, respectively.

## 4. Discussion

### 4.1. Evolution of Ecological Security

The ecological security in the PRD decreased with fluctuation during the study period, mainly due to the decline in the indicators in the pressure layer, consistent with the conclusions of Fan et al. [31]. Since 1990, the PRD has experienced a rapid increase in the economy and population, thus causing a sharp increase in demand for varied ecosystem services. In addition to the water consumption per unit area and grain consumption per capita, all of the other indicators in the pressure layer showed a significantly increased trend. CO_2_ emissions per unit area increased from 0.127 t/km^2^ in 1990 to 1.106 t/km^2^ in 2015, and per capita GDP increased from 4029.4 RMB/capita to 57,166.8 RMB/capita, which increased by 7.69 and 13.19 times, respectively. Furthermore, the population density and the proportion of construction land to the total land area increased by 145% and 157%, respectively. In the state layers, the per capita share of grain, per capita forest land area, and proportion of good habitat area became worse during the study period due to urban expansion and infrastructure construction, decreasing by 79.8%, 60.0%, and 5.3%, respectively. The agricultural mechanization level in the response layers decreased by 12.2%, mainly due to the arable land in the plain areas being occupied by construction land and the arable land in the hilly regions being unsuitable for agricultural machines. The proportion of ecological land in the response layer decreased by 1.6%. These indicators caused the ecological security decline mainly due to the increase in population and economy and the decrease in ecological land area.

There is some literature related to the ecological security of PRD. For example, Li et al. [19,32] examined the ecological security pattern based on the PSR model and BP-DEMALTE models. Hu et al. [13] analyzed the spatial differentiation of ecological security based on the indices embedded in ecosystem services. Due to the methods and variables being different, the results of these studies are not comparable. However, the change trends of ecological security showed a decreasing trend.

### 4.2. Evolution of Obstacle Factors and Policy Implications

The evolution path of obstacle factors in the PRD showed clear rules. The five key obstacle factors were derived initially from the state and response layers, then mainly from the pressure layer. The study periods were classified into three stages according to the evolution characteristics. In the first stage, between 1990 and 2000, the degree of the key obstacle factors from the state layer and pressure layer decreased while the response layer increased, indicating that the government’s response has lagged behind the environmental issues. In the second stage, the key obstacle degree in the pressure layer increased quickly. In contrast, the degree in response layers and state layers decreased from 2000 to 2005, showing that the pressure on the ecosystem increased. In the third stage, the obstacle degree in the pressure and state layers further increased. However, the indicators from the response layer were missed from 2005 to 2015, indicating that the indicators related to ecosystem service demand and supply caused the decrease in security. During the study period, the obstacle degree of the indicators from the response layer decreased with fluctuation caused by the sharp increase in the ecosystem service demand and the measures taken by governments. The evolution path of the five top key obstacle factors indicated that ecosystems have been under greater pressure in recent years than before. During the study period, the accumulative degree of the five top obstacle factors was above 45%. Therefore, governments should grasp the key indicators to improve the ecological security state.

For the foreseeable future, with rapid economic development and population expansion, ecosystem service demand will increase sharply, putting tremendous pressure on the ecosystems. Therefore, the PRD should take measures to improve the ecosystem quality and alleviate the pressure on the ecosystems brought by humans. First, the PRD should further promote its structural upgrade, develop a circular economy, reduce fossil fuel use, and prohibit high carbon emission industries. These measures are related to the improvement in P2, R2, and R6. Second, it needs to strengthen the optimization of land use and promote territorial space planning as well as identify and protect the source area of the key ecosystem services, prohibiting the occupation of ecological land by construction land and strictly observing the ecological protection red line (improving the indicators of P5, S1, S2, S4, S5, and R3). Third, the population can be controlled and the population density reduced by constructing a satellite city surrounding urban agglomeration (pertaining to P4, S1, and S4). Finally, the residents should develop low-carbon and sustainable consumption lifestyles, placing less pressure on the ecosystems.

### 4.3. Limitations and Prospects

This study brought the supply and demand of different ecosystem services into the evaluation index system of ecological security based on the PSR model, providing more objective and accurate decision-making information for sustainable development, however, some limitations did exist. First, we only considered five key ecosystem services in the PRD, neglecting other ecosystem services (water purification et al.) due to data availability. Therefore, other ecosystem services should be embedded into the PSR model. Second, we only evaluated ecological security in the PRD from 1990 to 2015 and did not analyze the change trends in the most recent years. Although the evolution trends of ecological security were depicted, ecological security close to the recent period should be evaluated to provide more practical policy implications. Third, scholars have classified the ecological security level according to the corresponding threshold defined in the research, so the classification of ecological security levels and thresholds is still unclear [33], thus, the mechanism of the ecological transformation process should be strengthened in further study.

## 5. Conclusions

Evaluating regional ecological security is one of the hot spots in social and scientific research, which is the significance of sustainable development. This study assessed the ecological security combined with the ecosystem service supply and demand with the PSR model. We identified the key obstacle factors based on the obstacle degree model in the PRD from 1990 to 2015.

In addition to grain production and habitat quality, soil retention, carbon sequestration, and water yield increased with fluctuation. The grain demand, carbon emissions, and water demand increased sharply by 10.1%, 769.4%, and 17.5%, respectively. Only the water supply met the human demands, while the budget for grain supply and carbon sequestration reached 2.86 and 506.13 million tons, respectively. The ecosystem service supply areas were distributed in the low hills, while the demand regions were in the low plain areas. The state layer had the most significant impacts on ecological security, followed by the response layer and pressure layer. The index of the state and response layers increased with fluctuation from 1990 to 2015, increasing by 0.006 and 0.037, respectively. The pressure index decreased by about 0.076, indicating increased pressure on the ecosystem. Change trends of these three indices resulted in the decline in the ecological security index in volatility, denoting that ecological security showed an inevitable deterioration in the PRD. During 1990 and 2000, the obstacle factors were mainly from the state and response layers, mainly from the pressure layer during 2005 and 2015. The accumulative degree of the five top obstacle factors was above 45%. Therefore, governments should grasp the key indicators to improve the ecological security state. This study provides a theoretical basis and scientific information for sustainable development in the PRD and other regions.

## Figures and Tables

**Table 1:** Change trends of different land use in the Pearl River Delta from 1990 to 2015.

Year	Arable Land (Unit: km^2^)	Forest Land (Unit: km^2^)	Grassland (Unit: km^2^)	Water Bodies (Unit: km^2^)	Construction Land (Unit: km^2^)	Other Land (Unit: km^2^)
1990	15,775.79	29,985.38	1097.10	3382.41	2908.48	171.26
1995	13,084.41	30,218.52	1392.18	3832.62	4654.75	143.88
2000	14,301.38	29,699.66	1049.95	3999.70	4168.85	102.85
2005	12,998.94	29,333.94	980.14	3866.22	6030.80	112.87
2010	12,574.05	29,187.31	945.08	3780.23	6722.40	114.94
2015	12,420.10	28,788.97	1072.50	3458.54	7482.80	104.41

**Table 2:** The methods and main references to evaluate ecosystem services.

Ecosystem Service	Methods	Algorithms ^1^	Reference
Grain production	VCI	${\mathrm{GP}}_{i}={\mathrm{GP}}_{t}\times \frac{{\mathrm{VCI}}_{i}}{\sum_{i=1}^{n}{\mathrm{VCI}}_{i}}$	[20]
Soil conservation	RUSLE	$A_{c}=R\times K\times \mathrm{LS}\times \left(1-C\times P\right)$	[21]
Carbon sequestration	CASA	$\mathrm{NPP}\left(x,t\right)=\mathrm{APRA}\left(x,t\right)\times \varepsilon \left(x,t\right)$	[22]
Water yield	Water balance model	WY=PPT−ET±S≈PPT−ET	[23]
Habitat quality	InVEST	$Q_{\mathrm{xj}}=H_{j}\left(1-\left(\frac{D_{\mathrm{xj}}^{z}}{D_{\mathrm{xj}}^{z}+k^{z}}\right)\right)$	[24]

^1^ The meanings of the different variables and specific calculation formulas can be found in the corresponding references.

**Table 3:** The evaluation index system and their weights of ecological security for the Pearl River Delta.

Dimensions	Index	Code	Unit	Attributes	Weight
Pressure 0.321	Grain consumption per capita	P1	kg/capita	−	0.0519
	CO_2_ emissions per unit area	P2	t/km^2^	−	0.0536
	Water consumption per unit area	P3	10^4^ m^3^/km^2^	−	0.0482
	Population density	P4	capita/km^2^	−	0.0523
	Proportion of construction land area	P5	%	−	0.0530
	GDP per capita	P6	RMB/capita	−	0.0613
State 0.347	Per capita share of grain	S1	kg/capita	+	0.0481
	Carbon sequestration per unit area	S2	tons/km^2^	+	0.0601
	Water yield per unit area	S3	10^4^ m^3^/km^2^	+	0.0630
	Forest area per capita	S4	m^2^/capita	+	0.0489
	Proportion of good habitat area	S5	%	+	0.0590
	Soil retention intensity	S6	tons/km^2^	+	0.0681
Response 0.332	Agricultural mechanization level	R1	kw/km^2^	+	0.0534
	Proportion of coal consumption	R2	%	−	0.0774
	Proportion of ecological land	R3	%	+	0.0454
	Wastewater treatment rate	R4	%	+	0.0496
	Investment in anti-pollution projects as percentage of GDP.	R5	%	+	0.0516
	Proportion of the tertiary industry in GDP.	R6	%	+	0.0551

**Table 4:** Different ecosystem services in the Pearl River Delta from 1990 to 2015.

Ecosystem Service	1990	1995	2000	2005	2010	2015
Grain production (million tons)	6.98	5.67	5.53	3.80	3.22	3.47
Soil retention (billion tons)	24.30	36.58	39.07	41.13	37.32	42.13
Carbon sequestration (million tons)	91.45	74.32	111.43	92.60	98.38	101.59
Water yield (billion tons)	41.8	56.2	58.5	71.6	65.0	63.6
Habitat quality	0.7738	0.7679	0.7670	0.7465	0.7365	0.7234

**Table 5:** Demand of the different ecosystem services in the Pearl River Delta from 1990 to 2015.

Ecosystem Service	1990	1995	2000	2005	2010	2015
Grain demand (million tons)	5.75	6.22	7.88	7.14	7.27	6.33
Carbon emission (million tons)	69.90	197.43	245.88	444.55	502.26	607.72
Water demand (billion tons)	19.42	19.53	26.87	33.56	27.41	22.82

## Data Availability

The data presented in this study are available on request from the corresponding author.
